# Lu-atom-ordered oxonitridoaluminosilicate Ba_0.9_Ce_0.1_LuAl_0.2_Si_3.8_N_6.9_O_0.1_

**DOI:** 10.1107/S2056989020013158

**Published:** 2020-10-06

**Authors:** Rayko Simura, Hisanori Yamane

**Affiliations:** a Institute of Multidisciplinary Research for Advanced Materials, Tohoku, University, 2-1-1 Katahira, Aoba-ku, Sendai, 980-8577, Japan

**Keywords:** BaLuSi_4_N_7_, crystal structure, oxynitride

## Abstract

A single crystal of Ba_0.9_Ce_0.1_LuAl_0.2_Si_3.8_N_6.9_O_0.1_ (barium cerium lutetium aluminosilicate nitride oxide) was obtained by heating a mixed powder of Ba_3_N_2_, Si_3_N_4_, Al, Lu_2_O_3_, and CeO_2_ at 2173 K for 1 h under N_2_ gas at 0.85 MPa. X-ray single-crystal structure analysis revealed that the title oxynitride is hexa­gonal (lattice constants: *a* = 6.0378 (5) Å, *c* = 9.8133 (9) Å; space group: *P*6_3_ *mc*) and isostructural with BaYbSi_4_N_7_. (Ba,Ce) and Lu atoms occupy twelvefold and sixfold coordination sites, respectively.

## Abstract 2

A single crystal of Ba_0.9_Ce_0.1_LuAl_0.2_Si_3.8_N_6.9_O_0.1_ (barium cerium lutetium aluminosilicate nitride oxide) was obtained by heating a mixed powder of Ba_3_N_2_, Si_3_N_4_, Al, Lu_2_O_3_, and CeO_2_ at 2173 K for 1 h under N_2_ gas at 0.85 MPa. X-ray single-crystal structure analysis revealed that the title oxynitride is hexa­gonal and isostructural with BaYbSi_4_N_7_. (Ba,Ce) and Lu atoms occupy twelvefold and sixfold coordination sites, respectively.

## Chemical context   

Huppertz & Schnick (1997*b* ▸) determined the hexa­gonal crystal structures of two isotypic nitrides, SrYbSi_4_N_7_ [*a* = 5.9880 (3) Å, *c* = 9.7499 (9) Å] and BaYbSi_4_N_7_ [*a* = 6.0307 (2) Å, *c* = 9.8198 (4) Å] with space group *P*6_3_ *mc* (*Z* = 2), by single-crystal X-ray diffraction (XRD). In the crystal structure of BaYbSi_4_N_7_, the Ba, Yb, and Si atoms are coord­inated by twelve, six, and four N atoms of an anti­cubocta­hedron, octa­hedron, and a tetra­hedron, respectively. A three-dimensional framework of SiN_4_ tetra­hedra is formed by sharing vertex N atoms, and the inter­spaces of the framework are occupied by Ba and Yb atoms. N atoms at the N1 and N2 sites bond to two Si atoms, and N atoms at the N3 site are surrounded by four Si atoms. Such a high coordination number for the N3 site is characteristic of the crystal structures of SrYbSi_4_N_7_ and BaYbSi_4_N_7_ (Huppertz & Schnick, 1997*b* ▸).

Other nitrides having the same structure type have been synthesized by substitution of Ca and/or other rare-earth (*R*) atoms for Sr, Ba, and Yb atoms. The crystal structure of SrYSi_4_N_7_ (*a* = 6.0160 (1) Å, *c* = 9.7894 (1) Å) was clarified by powder X-ray diffraction (pXRD) (Li, Fang, *et al.*, 2004 ▸). Some nitrides doped with Eu^2+^, such as Ba_0.99_Eu_0.01_YSi_4_N_7_ [*a* = 6.0275 (6) Å, *c* = 9.880 (1) Å], Sr_0.99_Eu_0.01_YSi_4_N_7_ [*a* = 6.0269 (7) Å, *c* = 9.878 (1)] , and Ca_0.99_Eu_0.01_YSi_4_N_7_ [*a* = 5.9866 (5) Å, *c* = 9.800 (1) Å] (Li, Fang, *et al.*, 2004 ▸; Porob *et al.*, 2012 ▸), have also been reported. Oxynitrides Sr*R*(Si,Al)_4_(N,O)_7_ and Ba*R*(Si,Al)_4_(N,O)_7_ (*R* = Ho, Er, Tm, Yb; Lieb *et al.*, 2007 ▸), in which the Si and N atoms are partly replaced by Al and O atoms, have also been synthesized. The crystal structures of the aforementioned compounds were found to be isotypic with SrYbSi_4_N_7_ and BaYbSi_4_N_7_. The alkaline-earth (*A*) atoms of Ca, Sr, or Ba are ordered at the anti­cubocta­hedral (*a*) site of twelvefold coordination of N or O atoms, and the *R* atoms are located at the octa­hedral (*o*) site of sixfold coordination of N or O atoms. However, the crystal structures of BaLuSi_4_N_7_ [*a* = 6.02185 (2) Å, *c* = 9.81219 (7) Å] and SrLuSi_4_N_7_ [*a* = 6.02113 (2) Å, *c* = 9.80105 (7) Å] were analyzed by the Rietveld method for pXRD patterns using a disordered model in which both Ba/Sr and Lu atoms were statistically located at the *a* and *o* sites with the same occupancy of 0.5 (Park *et al.*, 2012 ▸).

**Figure 1:**
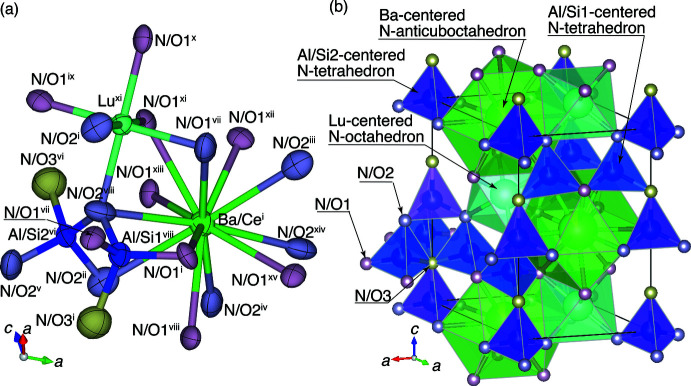
(*a*) Arrangement of cation-centered N/O atoms and (*b*) the crystal structure illustrated with cation-centered N/O-coordinated polyhedra for Ba_0.90_Ce_0.10_LuSi_3.80_Al_0.20_N_6.90_O_0.10_. Symmetry codes: (i) *x*, *y*, *z*; (ii) *x* − 1, *y*, *z*; (iii) *x*, *y* + 1, *z*; (iv) −*y*, *x* − *y*, *z*); (v) −*y*, *x* − *y* − 1, *z*; (vi) −*x*, −*y*, *z* + 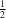); (vii) −*y* + 1, *x* − *y*, *z*); (viii) −*x* + *y* + 1, −*x* + 1, *z*; (ix) *y*, −*x* + *y*, *z* + 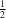; (x) *x* − *y* + 1, *x*, *z* + 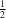; (xi) −*x* + 1, −*y* + 1, *z* + 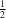; (xii) *y*, −*x* + *y* + 1, *z* + 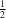; (xiii) *x* − *y*, *x*, *z* + 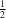; (xiv) −*x* + *y* + 1, −*x* + 2, *z*; (xv) −*y* + 1, *x* − *y* + 1, *z*.

During our materials survey of novel Ce-doped phosphors in the Ba–Lu–Si–N system, small numbers of needle-like single crystals of 10 μm in diameter and 60 μm in length (at maximum) were grown at the contact surface between the BN crucible and an aggregate of fine particles consisting of amorphous and crystalline materials. The powder XRD pattern of the crystalline materials were indexed by the similar lattice constants as that of the needle-like crystals. Electron-probe microanalysis (EPMA) performed at 12 points on one of the needle-like single crystals gave a composition of Ce:Ba:Lu:Si:Al:N:O = 0.8 (2):7.6 (5):7.6 (6):29.6 (20):1.6 (4):49 (3):4(1) in weight percent (total mass was normalized to 100 mass%). The lower precision of the N and O contents was due to the lower energy of the characteristic X-rays of these light elements. The molar ratio obtained from the composition was Ce:Ba:Lu:Si:Al:N:O = 0.1 (3):0.99 (7):0.99 (8):3.9 (3):0.21 (5):6.4 (4):0.5 (2) (total sum 13), and the composition of the single crystal was regarded to be Ce_0.1_Ba_0.9_Lu_1.0_Si_3.8_Al_0.2_N_6.9_O_0.1_ by assuming Ce atoms situated at the *a* site with Ba atoms. The XRD spots from the crystal were indexed with hexa­gonal lattice constants of *a* = 6.0378 (5) Å and *c* = 9.8133 (9) Å (Table 1 ▸), which were approximately the same as those reported for BaLuSi_4_N_7_ (Park *et al.*, 2012 ▸) within differences of 0.1 and 0.2%, respectively. Initially, a structure refinement of Ce_0.1_Ba_0.9_Lu_1.0_Si_3.8_Al_0.2_N_6.9_O_0.1_ was carried out with a disordered model of (Ce_0.1_Ba_0.4_Lu_0.5_)(Ba_0.5_Lu_0.5_)Al_0.05_Si_0.95_)_4_(N_0.99_O_0.01_)_7_, in which the Ce, Ba, and Lu atoms were at the *a* site with a ratio of 0.1:0.4:0.5 and Ba and Lu atoms were at the *o* site with a 0.5:0.5 ratio, in accordance with the structure model of BaLuSi_4_N_7_ (Park *et al.*, 2012 ▸). The *R* value of refinement was 4.2%, and residual electron densities of 5.52 and −3.46 e Å^−3^ were observed at 0.89 and 1.67 Å, respectively, from the *a* site and the N/O site (Table 2 ▸). Refinement using the ordered model of (Ce_0.1_Ba_0.9_)(Lu)(Al_0.05_Si_0.95_)_4_(N_0.99_O_0.01_)_7_, in which the Ba and Ce atoms are at the *a* site with a ratio of 0.9:0.1 and Lu atoms fully occupy the *o* site, yielded an *R* value of 2.2% with residual electron densities of 1.70 and −1.40 e Å^−3^ (Table1). As a consequence, the Ba and Lu atoms in Ba_0.9_Ce_0.1_LuAl_0.2_Si_3.8_N_6.9_O_0.1_ were clarified to be ordered at the *a* and *o* sites, respectively (Fig. 1 ▸).

## Structural commentary   

The inter­atomic distances of Ba/Ce—N/O for Ba_0.9_Ce_0.1_LuAl_0.2_Si_3.8_N_6.9_O_0.1_ are 2.975 (10) Å × 3, 3.0236 (5) Å × 4, 3.0236 (5) Å × 2, and 3.052 (10) Å × 3, which are comparable with the Ba/Lu—N distances for the *a* site of BaLuSi_4_N_7_ (2.975 Å × 3, 3.0372 Å × 3, 3.038 Å × 3, 3.0783 Å × 3) reported by Park *et al.* (2012 ▸). Lu—N/O distances in the title compound are 2.271 (10) Å × 3 and 2.312 (9) Å × 3, which are 0.139 Å shorter than the Ba/Lu—N distances (2.414 Å × 3, 2.451 Å × 3) for the *o* site of BaLuSi_4_N_7_.

The Al/Si1—N/O distances are 1.701 (9) Å × 3 and 1.85 (2) Å, and the Al/Si2—N/O distances are 1.738 (9) Å, 1.743 (6) Å × 2, and 1.954 (7) Å. These distances are consistent with those of Si—N (1.705 Å × 3, 1.887 Å and 1.724 Å, 1.721 Å × 2, 1.962 Å) for BaYbSi_4_N_7_ (Huppertz & Schnick, 1997*b* ▸) but 0.07–0.2 Å longer than those of Si1–N (1.478 Å × 3, 1.776 Å) and Si2—N (1.671, 1.673, 1.889, 1.937 Å) reported for BaLuSi_4_N_7_ by Park *et al.* (2012 ▸), although the lattice constants of Ba_0.9_Ce_0.1_LuAl_0.2_Si_3.8_N_6.9_O_0.1_ and BaLuSi_4_N_7_ are similar, as previously mentioned. The average distances of Al/Si2—N/O and Si2—O of 1.792 and 1.782 Å, respectively, are slightly longer than those of Al/Si1—N/O (1.741 Å) and Si1—N (1.750 Å). The ^IV^Si^4+^—^IV^N^3−^ and ^IV^Al^3+^—^IV^N^3−^ distances calculated with the effective ionic radius for nitrides (^IV^Si^4+^ = 0.29, ^IV^Al^3+^ = 0.41 Å, ^IV^N^3−^ = 1.46 Å; Baur, 1987 ▸) are 1.75 and 1.87 Å, respectively, which are similar to the Si—N and Al/Si—N/O distances of BaYbSi_4_N_7_ and Ba_0.9_Ce_0.1_LuAl_0.2_Si_3.8_N_6.9_O_0.1_. The bond-valence sum (BVS) (Brown & Altermatt, 1985 ▸) for the Lu site of Ba_0.9_Ce_0.1_LuAl_0.2_Si_3.8_N_6.9_O_0.1_ was calculated to be 3.07 with a bond-valence parameter of Lu—N (*r*_0_ = 2.046, *b* = 0.37) reported by Brese & O’Keeffe (1991 ▸), in good agreement with the valence of Lu^3+^. The BVS with a parameter of Ba—N [*r*_0_ = 2.47; Brese and O’Keeffe (1991 ▸)] is 2.73, which is greater than the valence of Ba^2+^. The BVSs of Al/Si1 and Al/Si2 with the parameter of Si–N (*r*_0_ = 1.77, *b* = 0.37) are 4.39 and 3.87, respectively.

## Database survey   

The Inorganic Crystal Structure Database (ICSD) includes some records of BaYbSi_4_N_7_-type nitrides and oxynitrides that include alkaline-earth and rare-earth elements: BaYbSi_4_N_7_ and SrYbSi_4_N_7_ by Huppertz & Schnick (1997*b* ▸) and SrYSi_4_N_7_ by Li, Fang *et al.* (2004 ▸). EuYbSi_4_N_7_ and EuYSi_4_N_7_ (Huppertz & Schnick, 1997*a* ▸; Li, Fang *et al.*, 2004 ▸) are isostructural with BaYbSi_4_N_7_ but do not include an alkaline-earth metal element.

Oxynitrides in which Si and N atoms were partly replaced with Al and O atoms, respectively, have also been reported: BaYb(Si,Al)_4_(O,N)_7_ (Vinograd *et al.*, 2007 ▸), BaEr(Si,Al)_4_(O,N)_7_, BaHo(Si,Al)_4_(O,N)_7_, BaTm(Si,Al)_4_(O,N)_7_, BaYb(Si,Al)_4_(O,N)_7_, SrEr(Si,Al)_4_(O,N)_7_, SrHo(Si,Al)_4_(O,N)_7_, SrTm(Si,Al)_4_(O,N)_7_, SrYb(Si,Al)_4_(O,N)_7_, EuEr(Si,Al)_4_(O,N)_7_, EuHo(Si,Al)_4_(O,N)_7_, EuTm(Si,Al)_4_(O,N)_7_, and EuYb(Si,Al)_4_(O,N)_7_ (Lieb *et al.*, 2007 ▸).

First-principles calculations of the electronic structures of SrYSi_4_N_7_ and BaYSi_4_N_7_ have been reported (Fang *et al.*, 2003 ▸). Moreover, numerous researchers have investigated the luminescence of oxynitrides and nitrides doped with Ce and Eu, including Ce^3+^-BaYSi_4_N_7_, Eu^2+^-BaYSi_4_N_7_ (Li, deWith *et al.*, 2004 ▸), Ce^3+^-SrYSi_4_N_7_, Eu^2+^-SrYSi_4_N_7_ (Li, Fang *et al.*, 2004 ▸), Eu^2+^-(Ca,Sr, or Ba)YSi_4_N_7_, Eu^2+^-(Ca,Sr, or Ba)Y(Si,Al)_4_(N,O)_7_ (Kurushima *et al.*, 2010 ▸), Eu^2+^-(Ca, Sr, or Ba)(Sc, Y, or La)Si_4_N_7_ (Horikawa *et al.*, 2012 ▸), Eu^2+^-(Ca,Sr, or Ba)Y(Y, La, or Lu)Si_4_N_7_ (Park *et al.*, 2012 ▸), and Eu^2+^-SrScSi_4_(N,O)_7_ (Porob *et al.*, 2012 ▸).

## Synthesis and crystallization   

Powdered Si_3_N_4_ (Ube Industries Ltd., UBE-SN-E10, 95+%), Ba_3_N_2_ (Materion Corp., ∼20 mesh 99.7%), Al (Rare Metallic, ∼200 mesh, 99.9%), Lu_2_O_3_ (Nippon Yttrium Co. Ltd., 99.999%), CeO_2_ (Shin-Etsu Chemical Co. Ltd., 99.99%) were weighed out in an Si:Ba:Lu:Al:Ce molar ratio of 3.25:1:1:0.25:0.04 in an Ar-filled glove box (MBRAUN; [O_2_] and [H_2_O] < 1 ppm). The weighed powders were mixed in an agate mortar, and a disk-shaped pellet with a diameter of 10 mm was formed with a die in an Ar gas-filled glove box. The pellet was placed in a BN crucible (Showa Denko, K. K., 99.5%) with an 18 mm inner diameter and 20 mm height, and a BN lid was placed on it. The BN crucible was heated to 1200°C for 1 h under vacuum using a carbon furnace (VESTA, Shimadzu Industrial Systems Co., Ltd.), and the temperature was maintained at 1200 °C for 1 h. N_2_ gas (Taiyo Nippon Sanso Corp., 99.9995+%) was introduced into the furnace to a pressure of 0.85 MPa, and the furnace was then heated to 1900°C for 25 min. After the temperature and the N_2_ gas pressure were maintained for 1 h, the sample was cooled to 1200°C for 25 min. The heater power was then cut off. After the furnace reached room temperature, the crucible was removed from the furnace. The chemical composition of the single crystal was analyzed by EPMA (JEOL JXA-8200).

## Refinement   

Crystal data and the data collection details are summarized in Table 1 ▸, and the structural refinement details are reported in Table 2 ▸. Ordered and disordered models were investigated, and the best result was obtained using an ordered model in which the Ce/Ba mixed site and Lu site are located at the *a* site and *o* site, respectively. Because the *R* and *S* values were not affected by refinement with ordered models of Al and Si atoms and N and O atoms, the occupancies of the Al/Si and N/O sites were fixed at 0.05/0.95 and 0.99/0.01, respectively. Final refinement was carried out with anisotropic displacement parameters.

## Supplementary Material

Crystal structure: contains datablock(s) I. DOI: 10.1107/S2056989020013158/ru2072sup1.cif

Structure factors: contains datablock(s) I. DOI: 10.1107/S2056989020013158/ru2072Isup2.hkl

CCDC reference: 2034534

Additional supporting information:  crystallographic information; 3D view; checkCIF report

## Figures and Tables

**Table 1:** Experimental details

Crystal data	
Chemical formula	Ba_0.9_Ce_0.1_LuAl_0.2_Si_3.8_N_6.9_O_0.1_
*M*_r_	1045.99
Crystal system, space group	Hexagonal, *P*6_3_ *m* *c*
Temperature (K)	301
*a*, *c* (Å)	6.0378 (5), 9.8133 (9)
*V* (Å^3^)	309.82 (6)
*Z*	1
Radiation type	Mo *K*α
μ (mm^−1^)	22.95
Crystal size (μm)	0.13 × 0.07 × 0.02

Data collection	
Diffractometer	Bruker D8 QUEST
Absorption correction	Multi-scan (*SADABS*; Bruker, 2018 ▸)
*T*_min_, *T*_max_	0.37, 0.68
No. of measured, independent and observed [*I* > 2σ(*I*)] reflections	2818, 395, 381
*R*_int_	0.059
(sin θ/λ)_max_ (Å^−1^)	0.713

Refinement	
*R*[*F* ^2^ > 2σ(*F* ^2^)], *wR*(*F* ^2^), *S*	0.022, 0.055, 1.04
No. of reflections	395
No. of parameters	33
No. of restraints	1
Δρ_max_, Δρ_min_ (e Å^−3^)	1.70, −1.40
Absolute structure	Refined as an inversion twin
Absolute structure parameter	0.10 (3)

Computer programs: Instrument Service (Bruker, 2018 ▸), *APEX3* (Bruker, 2018 ▸), *SAINT* (Bruker, 2018 ▸), *SHELXT2014/5* (Sheldrick, 2015*a* ▸), *SHELXL2014/7* (Sheldrick, 2015*b* ▸), *VESTA* (Momma & Izumi, 2011 ▸), pubCIF (Westrip, 2010 ▸).

**Table 2:** Disordered model (Ce_0.1_Ba_0.4_Lu_0.5_)(Ba_0.5_Lu_0.5_) (Al_0.05_Si_0.95_)_4_ (N_0.99_O_0.01_)_7_

Refinement	
*R*[*F* ^2^ > 2*σ*(*F* ^2^)], *wR*(*F* ^2^), *S*	0.042, 0.115, 1.32
No. of reflections	395
No. of parameters	39
No. of restraints	1
*Δρ_max_*, *Δρ_min_* (e Å^−3^)	5.52, −3.46
Absolute structure	Refined as an inversion twin
Absolute structure parameter	0.12 (9)

## supplementary crystallographic information

### Crystal data

**Table:**

Ba_0.9_Ce_0.1_LuAl_0.2_Si_3.8_N_6.9_O_0.1_	*D*_x_ = 5.606 Mg m^−^^3^
*M**_r_* = 1045.99	Mo *K*α radiation, λ = 0.71073 Å
Hexagonal, *P*6_3_*m**c*	Cell parameters from 127 reflections
*a* = 6.0378 (5) Å	θ = 4.2–30.8°
*c* = 9.8133 (9) Å	µ = 22.95 mm^−^^1^
*V* = 309.82 (6) Å^3^	*T* = 301 K
*Z* = 1	Block, colorless
*F*(000) = 464	0.13 × 0.07 × 0.02 mm

### Data collection

**Table:**

Bruker D8 QUEST diffractometer	381 reflections with *I* > 2σ(*I*)
Detector resolution: 7.3910 pixels mm^-1^	*R*_int_ = 0.059
ω and σcans	θ_max_ = 30.5°, θ_min_ = 3.9°
Absorption correction: multi-scan (SADABS; Bruker, 2018)	*h* = −7→8
*T*_min_ = 0.37, *T*_max_ = 0.68	*k* = −8→8
2818 measured reflections	*l* = −14→14
395 independent reflections	

### Refinement

**Table:**

Refinement on *F*^2^	*w* = 1/[σ^2^(*F*_o_^2^) + (0.0313*P*)^2^ + 0.7267*P*] where *P* = (*F*_o_^2^ + 2*F*_c_^2^)/3
Least-squares matrix: full	(Δ/σ)_max_ < 0.001
*R*[*F*^2^ > 2σ(*F*^2^)] = 0.022	Δρ_max_ = 1.70 e Å^−^^3^
*wR*(*F*^2^) = 0.055	Δρ_min_ = −1.40 e Å^−^^3^
*S* = 1.04	Extinction correction: SHELXL-2014/7 (Sheldrick 2015b), Fc^*^=kFc[1+0.001xFc^2^λ^3^/sin(2θ)]^-1/4^
395 reflections	Extinction coefficient: 0.0052 (16)
33 parameters	Absolute structure: Refined as an inversion twin.
1 restraint	Absolute structure parameter: 0.10 (3)

### Special details

**Table:**

Geometry. All esds (except the esd in the dihedral angle between two l.s. planes) are estimated using the full covariance matrix. The cell esds are taken into account individually in the estimation of esds in distances, angles and torsion angles; correlations between esds in cell parameters are only used when they are defined by crystal symmetry. An approximate (isotropic) treatment of cell esds is used for estimating esds involving l.s. planes.
Refinement. Refined as a two-component inversion twin

### Fractional atomic coordinates and isotropic or equivalent isotropic displacement parameters (Å^2^)

**Table:**

	*x*	*y*	*z*	*U*_iso_*/*U*_eq_	Occ. (<1)
Lu1	0.3333	0.6667	0.07616 (4)	0.0074 (2)	
Ba2	0.3333	0.6667	0.44902 (10)	0.0099 (3)	0.9
Ce2	0.3333	0.6667	0.44902 (10)	0.0099 (3)	0.1
Si1	0.8275 (2)	0.1725 (2)	0.2646 (5)	0.0084 (5)	0.95
Al1	0.8275 (2)	0.1725 (2)	0.2646 (5)	0.0084 (5)	0.05
Si2	0.0000	0.0000	0.0006 (7)	0.0071 (9)	0.95
Al2	0.0000	0.0000	0.0006 (7)	0.0071 (9)	0.05
N1	0.5097 (8)	0.4903 (8)	0.2112 (11)	0.0087 (14)	0.9857
O1	0.5097 (8)	0.4903 (8)	0.2112 (11)	0.0087 (14)	0.0143
N2	0.8474 (8)	0.1526 (8)	0.4404 (9)	0.0123 (19)	0.9857
O2	0.8474 (8)	0.1526 (8)	0.4404 (9)	0.0123 (19)	0.0143
N3	0.0000	0.0000	0.1880 (18)	0.016 (3)	0.9857
O3	0.0000	0.0000	0.1880 (18)	0.016 (3)	0.0143

### Atomic displacement parameters (Å^2^)

**Table:**

	*U*^11^	*U*^22^	*U*^33^	*U*^12^	*U*^13^	*U*^23^
Lu1	0.0075 (3)	0.0075 (3)	0.0072 (4)	0.00374 (13)	0.000	0.000
Ba2	0.0098 (4)	0.0098 (4)	0.0101 (6)	0.00489 (18)	0.000	0.000
Ce2	0.0098 (4)	0.0098 (4)	0.0101 (6)	0.00489 (18)	0.000	0.000
Si1	0.0076 (8)	0.0076 (8)	0.0097 (10)	0.0035 (9)	−0.0002 (6)	0.0002 (6)
Al1	0.0076 (8)	0.0076 (8)	0.0097 (10)	0.0035 (9)	−0.0002 (6)	0.0002 (6)
Si2	0.0061 (10)	0.0061 (10)	0.009 (3)	0.0031 (5)	0.000	0.000
Al2	0.0061 (10)	0.0061 (10)	0.009 (3)	0.0031 (5)	0.000	0.000
N1	0.007 (2)	0.007 (2)	0.010 (3)	0.002 (2)	0.0010 (16)	−0.0010 (16)
O1	0.007 (2)	0.007 (2)	0.010 (3)	0.002 (2)	0.0010 (16)	−0.0010 (16)
N2	0.015 (3)	0.015 (3)	0.011 (5)	0.011 (4)	0.0010 (14)	−0.0010 (14)
O2	0.015 (3)	0.015 (3)	0.011 (5)	0.011 (4)	0.0010 (14)	−0.0010 (14)
N3	0.018 (5)	0.018 (5)	0.013 (8)	0.009 (3)	0.000	0.000
O3	0.018 (5)	0.018 (5)	0.013 (8)	0.009 (3)	0.000	0.000

### Geometric parameters (Å, º)

**Table:**

Lu1—O1^i^	2.271 (10)	Si1—Ba2^ix^	3.521 (2)
Lu1—N1^i^	2.271 (10)	Si1—Ba2^x^	3.521 (2)
Lu1—O1^ii^	2.271 (10)	Si2—O2^xi^	1.701 (9)
Lu1—N1^ii^	2.271 (10)	Si2—N2^xi^	1.701 (9)
Lu1—N1	2.271 (10)	Si2—O2^iv^	1.701 (9)
Lu1—O2^iii^	2.312 (9)	Si2—N2^iv^	1.701 (9)
Lu1—N2^iii^	2.312 (9)	Si2—O2^xii^	1.701 (9)
Lu1—O2^iv^	2.312 (9)	Si2—N2^xii^	1.701 (9)
Lu1—N2^iv^	2.312 (9)	Si2—N3	1.839 (19)
Lu1—O2^v^	2.312 (9)	Si2—Ba2^xiii^	3.5225 (11)
Lu1—N2^v^	2.312 (9)	Si2—Ba2^xiv^	3.5225 (11)
Ba2—N1	2.975 (10)	Si2—Ba2^v^	3.5226 (11)
Ba2—O1^ii^	2.975 (10)	N1—Al1^vii^	1.743 (6)
Ba2—N1^ii^	2.975 (10)	N1—Si1^vii^	1.743 (6)
Ba2—O1^i^	2.975 (10)	N1—Al1^vi^	1.743 (6)
Ba2—N1^i^	2.975 (10)	N1—Si1^vi^	1.743 (6)
Ba2—O2^vi^	3.0236 (5)	N1—Ba2^xiv^	3.052 (10)
Ba2—N2^vi^	3.0236 (5)	N2—Al2^xv^	1.701 (9)
Ba2—O2^vii^	3.0236 (5)	N2—Si2^xv^	1.701 (9)
Ba2—N2^vii^	3.0236 (5)	N2—Lu1^xv^	2.313 (9)
Ba2—O2^viii^	3.0237 (6)	N2—Ba2^ix^	3.0237 (6)
Ba2—N2^viii^	3.0237 (6)	N2—Ce2^ix^	3.0237 (6)
Si1—N2	1.738 (9)	N2—Ce2^x^	3.0237 (6)
Si1—O1^vi^	1.743 (6)	N2—Ba2^x^	3.0237 (6)
Si1—N1^vi^	1.743 (6)	N3—Al1^vi^	1.954 (7)
Si1—O1^vii^	1.743 (6)	N3—Si1^vi^	1.954 (7)
Si1—N1^vii^	1.743 (6)	N3—Al1^xvi^	1.954 (7)
Si1—O3^ix^	1.954 (7)	N3—Si1^xvi^	1.954 (7)
Si1—N3^ix^	1.954 (7)	N3—Si1^viii^	1.954 (7)
Si1—Al1^vii^	2.914 (4)	N3—Al1^viii^	1.954 (7)
Si1—Al1^vi^	2.914 (4)		
			
O1^i^—Lu1—N1^i^	0.0	N1^vi^—Si1—Al1^vi^	88.8 (2)
O1^i^—Lu1—O1^ii^	89.4 (3)	O1^vii^—Si1—Al1^vi^	33.3 (3)
N1^i^—Lu1—O1^ii^	89.4 (3)	N1^vii^—Si1—Al1^vi^	33.3 (3)
O1^i^—Lu1—N1^ii^	89.4	O3^ix^—Si1—Al1^vi^	143.1 (3)
N1^i^—Lu1—N1^ii^	89.4 (3)	N3^ix^—Si1—Al1^vi^	143.1 (3)
O1^ii^—Lu1—N1^ii^	0.0	Al1^vii^—Si1—Al1^vi^	60.0
O1^i^—Lu1—N1	89.4	N2—Si1—Ba2^ix^	59.17 (6)
N1^i^—Lu1—N1	89.4 (3)	O1^vi^—Si1—Ba2^ix^	57.6 (3)
O1^ii^—Lu1—N1	89.4	N1^vi^—Si1—Ba2^ix^	57.6 (3)
N1^ii^—Lu1—N1	89.4 (3)	O1^vii^—Si1—Ba2^ix^	149.6 (3)
O1^i^—Lu1—O2^iii^	90.2 (2)	N1^vii^—Si1—Ba2^ix^	149.6 (3)
N1^i^—Lu1—O2^iii^	90.2 (2)	O3^ix^—Si1—Ba2^ix^	100.5 (2)
O1^ii^—Lu1—O2^iii^	90.2 (2)	N3^ix^—Si1—Ba2^ix^	100.5 (2)
N1^ii^—Lu1—O2^iii^	90.2 (2)	Al1^vii^—Si1—Ba2^ix^	65.56 (4)
N1—Lu1—O2^iii^	179.5 (3)	Al1^vi^—Si1—Ba2^ix^	116.34 (4)
O1^i^—Lu1—N2^iii^	90.2 (2)	N2—Si1—Ba2^x^	59.17 (6)
N1^i^—Lu1—N2^iii^	90.2 (2)	O1^vi^—Si1—Ba2^x^	149.6 (3)
O1^ii^—Lu1—N2^iii^	90.2 (2)	N1^vi^—Si1—Ba2^x^	149.6 (3)
N1^ii^—Lu1—N2^iii^	90.2 (2)	O1^vii^—Si1—Ba2^x^	57.6 (3)
N1—Lu1—N2^iii^	179.5 (3)	N1^vii^—Si1—Ba2^x^	57.6 (3)
O2^iii^—Lu1—N2^iii^	0.0	O3^ix^—Si1—Ba2^x^	100.5 (2)
O1^i^—Lu1—O2^iv^	179.5 (3)	N3^ix^—Si1—Ba2^x^	100.5 (2)
N1^i^—Lu1—O2^iv^	179.5 (3)	Al1^vii^—Si1—Ba2^x^	116.34 (4)
O1^ii^—Lu1—O2^iv^	90.2 (2)	Al1^vi^—Si1—Ba2^x^	65.56 (4)
N1^ii^—Lu1—O2^iv^	90.2 (2)	Ba2^ix^—Si1—Ba2^x^	118.08 (12)
N1—Lu1—O2^iv^	90.2 (2)	O2^xi^—Si2—N2^xi^	0.0
O2^iii^—Lu1—O2^iv^	90.1 (3)	O2^xi^—Si2—O2^iv^	108.6 (4)
N2^iii^—Lu1—O2^iv^	90.1 (3)	N2^xi^—Si2—O2^iv^	108.6 (4)
O1^i^—Lu1—N2^iv^	179.5 (3)	O2^xi^—Si2—N2^iv^	108.6
N1^i^—Lu1—N2^iv^	179.5 (3)	N2^xi^—Si2—N2^iv^	108.6 (4)
O1^ii^—Lu1—N2^iv^	90.2 (2)	O2^iv^—Si2—N2^iv^	0.0
N1^ii^—Lu1—N2^iv^	90.2 (2)	O2^xi^—Si2—O2^xii^	108.6 (4)
N1—Lu1—N2^iv^	90.2 (2)	N2^xi^—Si2—O2^xii^	108.6 (4)
O2^iii^—Lu1—N2^iv^	90.1	O2^iv^—Si2—O2^xii^	108.6 (4)
N2^iii^—Lu1—N2^iv^	90.1 (3)	N2^iv^—Si2—O2^xii^	108.6 (4)
O2^iv^—Lu1—N2^iv^	0.0	O2^xi^—Si2—N2^xii^	108.6
O1^i^—Lu1—O2^v^	90.2 (2)	N2^xi^—Si2—N2^xii^	108.6 (4)
N1^i^—Lu1—O2^v^	90.2 (2)	O2^iv^—Si2—N2^xii^	108.6
O1^ii^—Lu1—O2^v^	179.5 (3)	N2^iv^—Si2—N2^xii^	108.6 (4)
N1^ii^—Lu1—O2^v^	179.5 (3)	O2^xii^—Si2—N2^xii^	0.0
N1—Lu1—O2^v^	90.2 (2)	O2^xi^—Si2—N3	110.3 (3)
O2^iii^—Lu1—O2^v^	90.1 (3)	N2^xi^—Si2—N3	110.3 (3)
N2^iii^—Lu1—O2^v^	90.1 (3)	O2^iv^—Si2—N3	110.3 (3)
O2^iv^—Lu1—O2^v^	90.1 (3)	N2^iv^—Si2—N3	110.3 (3)
N2^iv^—Lu1—O2^v^	90.1 (3)	O2^xii^—Si2—N3	110.3 (3)
O1^i^—Lu1—N2^v^	90.2 (2)	N2^xii^—Si2—N3	110.3 (3)
N1^i^—Lu1—N2^v^	90.2 (2)	O2^xi^—Si2—Ba2^xiii^	59.07 (3)
O1^ii^—Lu1—N2^v^	179.5 (3)	N2^xi^—Si2—Ba2^xiii^	59.07 (3)
N1^ii^—Lu1—N2^v^	179.5 (3)	O2^iv^—Si2—Ba2^xiii^	151.4 (4)
N1—Lu1—N2^v^	90.2 (2)	N2^iv^—Si2—Ba2^xiii^	151.4 (4)
O2^iii^—Lu1—N2^v^	90.1	O2^xii^—Si2—Ba2^xiii^	59.07 (3)
N2^iii^—Lu1—N2^v^	90.1 (3)	N2^xii^—Si2—Ba2^xiii^	59.07 (3)
O2^iv^—Lu1—N2^v^	90.1	N3—Si2—Ba2^xiii^	98.27 (11)
N2^iv^—Lu1—N2^v^	90.1 (3)	O2^xi^—Si2—Ba2^xiv^	151.4 (4)
O2^v^—Lu1—N2^v^	0.0	N2^xi^—Si2—Ba2^xiv^	151.4 (4)
N1—Ba2—O1^ii^	65.0	O2^iv^—Si2—Ba2^xiv^	59.07 (3)
N1—Ba2—N1^ii^	65.0 (3)	N2^iv^—Si2—Ba2^xiv^	59.07 (3)
O1^ii^—Ba2—N1^ii^	0.0	O2^xii^—Si2—Ba2^xiv^	59.07 (3)
N1—Ba2—O1^i^	65.0	N2^xii^—Si2—Ba2^xiv^	59.07 (3)
O1^ii^—Ba2—O1^i^	65.0 (3)	N3—Si2—Ba2^xiv^	98.27 (11)
N1^ii^—Ba2—O1^i^	65.0 (3)	Ba2^xiii^—Si2—Ba2^xiv^	117.97 (6)
N1—Ba2—N1^i^	65.0 (3)	O2^xi^—Si2—Ba2^v^	59.07 (3)
O1^ii^—Ba2—N1^i^	65.0	N2^xi^—Si2—Ba2^v^	59.07 (3)
N1^ii^—Ba2—N1^i^	65.0 (3)	O2^iv^—Si2—Ba2^v^	59.07 (3)
O1^i^—Ba2—N1^i^	0.0	N2^iv^—Si2—Ba2^v^	59.07 (3)
N1—Ba2—O2^vi^	57.1 (2)	O2^xii^—Si2—Ba2^v^	151.4 (4)
O1^ii^—Ba2—O2^vi^	87.01 (17)	N2^xii^—Si2—Ba2^v^	151.4 (4)
N1^ii^—Ba2—O2^vi^	87.01 (17)	N3—Si2—Ba2^v^	98.27 (11)
O1^i^—Ba2—O2^vi^	122.0 (3)	Ba2^xiii^—Si2—Ba2^v^	117.97 (6)
N1^i^—Ba2—O2^vi^	122.0 (3)	Ba2^xiv^—Si2—Ba2^v^	117.97 (6)
N1—Ba2—N2^vi^	57.1 (2)	Al1^vii^—N1—Si1^vii^	0.0
O1^ii^—Ba2—N2^vi^	87.01 (17)	Al1^vii^—N1—Al1^vi^	113.4 (6)
N1^ii^—Ba2—N2^vi^	87.01 (17)	Si1^vii^—N1—Al1^vi^	113.4 (6)
O1^i^—Ba2—N2^vi^	122.0 (3)	Al1^vii^—N1—Si1^vi^	113.4
N1^i^—Ba2—N2^vi^	122.0 (3)	Si1^vii^—N1—Si1^vi^	113.4 (6)
O2^vi^—Ba2—N2^vi^	0.0	Al1^vi^—N1—Si1^vi^	0.0
N1—Ba2—O2^vii^	57.1 (2)	Al1^vii^—N1—Lu1	123.3 (3)
O1^ii^—Ba2—O2^vii^	122.0 (3)	Si1^vii^—N1—Lu1	123.3 (3)
N1^ii^—Ba2—O2^vii^	122.0 (3)	Al1^vi^—N1—Lu1	123.3 (3)
O1^i^—Ba2—O2^vii^	87.01 (17)	Si1^vi^—N1—Lu1	123.3 (3)
N1^i^—Ba2—O2^vii^	87.01 (17)	Al1^vii^—N1—Ba2	92.8 (4)
O2^vi^—Ba2—O2^vii^	65.6 (3)	Si1^vii^—N1—Ba2	92.8 (4)
N2^vi^—Ba2—O2^vii^	65.6 (3)	Al1^vi^—N1—Ba2	92.8 (4)
N1—Ba2—N2^vii^	57.1 (2)	Si1^vi^—N1—Ba2	92.8 (4)
O1^ii^—Ba2—N2^vii^	122.0 (3)	Lu1—N1—Ba2	87.4 (2)
N1^ii^—Ba2—N2^vii^	122.0 (3)	Al1^vii^—N1—Ba2^xiv^	90.4 (3)
O1^i^—Ba2—N2^vii^	87.01 (17)	Si1^vii^—N1—Ba2^xiv^	90.4 (3)
N1^i^—Ba2—N2^vii^	87.01 (17)	Al1^vi^—N1—Ba2^xiv^	90.4 (3)
O2^vi^—Ba2—N2^vii^	65.6	Si1^vi^—N1—Ba2^xiv^	90.4 (3)
N2^vi^—Ba2—N2^vii^	65.6 (3)	Lu1—N1—Ba2^xiv^	86.8 (3)
O2^vii^—Ba2—N2^vii^	0.0	Ba2—N1—Ba2^xiv^	174.2 (4)
N1—Ba2—O2^viii^	87.01 (17)	Al2^xv^—N2—Si2^xv^	0.0
O1^ii^—Ba2—O2^viii^	57.1 (2)	Al2^xv^—N2—Si1	117.2 (5)
N1^ii^—Ba2—O2^viii^	57.1 (2)	Si2^xv^—N2—Si1	117.2 (5)
O1^i^—Ba2—O2^viii^	122.0 (3)	Al2^xv^—N2—Lu1^xv^	124.5 (5)
N1^i^—Ba2—O2^viii^	122.0 (3)	Si2^xv^—N2—Lu1^xv^	124.5 (5)
O2^vi^—Ba2—O2^viii^	54.4 (3)	Si1—N2—Lu1^xv^	118.3 (4)
N2^vi^—Ba2—O2^viii^	54.4 (3)	Al2^xv^—N2—Ba2^ix^	92.07 (17)
O2^vii^—Ba2—O2^viii^	119.924 (18)	Si2^xv^—N2—Ba2^ix^	92.07 (17)
N2^vii^—Ba2—O2^viii^	119.924 (18)	Si1—N2—Ba2^ix^	91.25 (18)
N1—Ba2—N2^viii^	87.01 (17)	Lu1^xv^—N2—Ba2^ix^	86.80 (17)
O1^ii^—Ba2—N2^viii^	57.1 (2)	Al2^xv^—N2—Ce2^ix^	92.07 (17)
N1^ii^—Ba2—N2^viii^	57.1 (2)	Si2^xv^—N2—Ce2^ix^	92.07 (17)
O1^i^—Ba2—N2^viii^	122.0 (3)	Si1—N2—Ce2^ix^	91.25 (18)
N1^i^—Ba2—N2^viii^	122.0 (3)	Lu1^xv^—N2—Ce2^ix^	86.80 (17)
O2^vi^—Ba2—N2^viii^	54.4	Ba2^ix^—N2—Ce2^ix^	0.0
N2^vi^—Ba2—N2^viii^	54.4 (3)	Al2^xv^—N2—Ce2^x^	92.07 (17)
O2^vii^—Ba2—N2^viii^	119.9	Si2^xv^—N2—Ce2^x^	92.07 (17)
N2^vii^—Ba2—N2^viii^	119.924 (18)	Si1—N2—Ce2^x^	91.25 (18)
O2^viii^—Ba2—N2^viii^	0.0	Lu1^xv^—N2—Ce2^x^	86.80 (17)
N2—Si1—O1^vi^	111.0 (4)	Ba2^ix^—N2—Ce2^x^	173.6 (3)
N2—Si1—N1^vi^	111.0 (4)	Ce2^ix^—N2—Ce2^x^	173.6 (3)
O1^vi^—Si1—N1^vi^	0.0	Al2^xv^—N2—Ba2^x^	92.07 (17)
N2—Si1—O1^vii^	111.0 (4)	Si2^xv^—N2—Ba2^x^	92.07 (17)
O1^vi^—Si1—O1^vii^	109.3 (6)	Si1—N2—Ba2^x^	91.25 (18)
N1^vi^—Si1—O1^vii^	109.3 (6)	Lu1^xv^—N2—Ba2^x^	86.80 (17)
N2—Si1—N1^vii^	111.0 (4)	Ba2^ix^—N2—Ba2^x^	173.6 (3)
O1^vi^—Si1—N1^vii^	109.3	Ce2^ix^—N2—Ba2^x^	173.6
N1^vi^—Si1—N1^vii^	109.3 (6)	Ce2^x^—N2—Ba2^x^	0.0
O1^vii^—Si1—N1^vii^	0.0	Si2—N3—Al1^vi^	112.6 (5)
N2—Si1—O3^ix^	105.7 (6)	Si2—N3—Si1^vi^	112.6 (5)
O1^vi^—Si1—O3^ix^	109.9 (4)	Al1^vi^—N3—Si1^vi^	0.0
N1^vi^—Si1—O3^ix^	109.9 (4)	Si2—N3—Al1^xvi^	112.6 (5)
O1^vii^—Si1—O3^ix^	109.9 (4)	Al1^vi^—N3—Al1^xvi^	106.2 (5)
N1^vii^—Si1—O3^ix^	109.9 (4)	Si1^vi^—N3—Al1^xvi^	106.2 (5)
N2—Si1—N3^ix^	105.7 (6)	Si2—N3—Si1^xvi^	112.6 (5)
O1^vi^—Si1—N3^ix^	109.9 (4)	Al1^vi^—N3—Si1^xvi^	106.2
N1^vi^—Si1—N3^ix^	109.9 (4)	Si1^vi^—N3—Si1^xvi^	106.2 (5)
O1^vii^—Si1—N3^ix^	109.9 (4)	Al1^xvi^—N3—Si1^xvi^	0.0
N1^vii^—Si1—N3^ix^	109.9 (4)	Si2—N3—Si1^viii^	112.6 (5)
O3^ix^—Si1—N3^ix^	0.0	Al1^vi^—N3—Si1^viii^	106.2
N2—Si1—Al1^vii^	96.0 (2)	Si1^vi^—N3—Si1^viii^	106.2 (5)
O1^vi^—Si1—Al1^vii^	33.3 (3)	Al1^xvi^—N3—Si1^viii^	106.2
N1^vi^—Si1—Al1^vii^	33.3 (3)	Si1^xvi^—N3—Si1^viii^	106.2 (5)
O1^vii^—Si1—Al1^vii^	88.8 (2)	Si2—N3—Al1^viii^	112.6 (5)
N1^vii^—Si1—Al1^vii^	88.8 (2)	Al1^vi^—N3—Al1^viii^	106.2 (5)
O3^ix^—Si1—Al1^vii^	143.1 (3)	Si1^vi^—N3—Al1^viii^	106.2 (5)
N3^ix^—Si1—Al1^vii^	143.1 (3)	Al1^xvi^—N3—Al1^viii^	106.2 (5)
N2—Si1—Al1^vi^	96.0 (2)	Si1^xvi^—N3—Al1^viii^	106.2 (5)
O1^vi^—Si1—Al1^vi^	88.8 (2)	Si1^viii^—N3—Al1^viii^	0.0 (2)

Symmetry codes: (i) −*y*+1, *x*−*y*+1, *z*; (ii) −*x*+*y*, −*x*+1, *z*; (iii) −*x*+1, −*y*+1, *z*−1/2; (iv) *y*, −*x*+*y*+1, *z*−1/2; (v) *x*−*y*, *x*, *z*−1/2; (vi) −*x*+*y*+1, −*x*+1, *z*; (vii) −*y*+1, *x*−*y*, *z*; (viii) *x*−1, *y*, *z*; (ix) *x*+1, *y*, *z*; (x) *x*, *y*−1, *z*; (xi) *x*−*y*−1, *x*−1, *z*−1/2; (xii) −*x*+1, −*y*, *z*−1/2; (xiii) *x*−*y*, *x*−1, *z*−1/2; (xiv) *x*−*y*+1, *x*, *z*−1/2; (xv) *x*−*y*+1, *x*, *z*+1/2; (xvi) −*y*, *x*−*y*−1, *z*.
